# Role of Laparotomy-based Parameters in Assessment of Optimal Primary Debulking Surgery and Long-term Outcomes in Patients with Stage IIIC Epithelial Ovarian Cancer

**DOI:** 10.7150/jca.32317

**Published:** 2020-01-01

**Authors:** Mingyi Zhou, Danbo Wang, Zaiqiu Long, Yong Zhang, Jing Liu

**Affiliations:** 1 Department of Gynecology, Cancer Hospital of China Medical University, Liaoning Cancer Hospital & Institute, Shenyang 110042, Liaoning Province, PR China.; 2 Department of Pathology, Cancer Hospital of China Medical University, Liaoning Cancer Hospital & Institute, Shenyang 110042, Liaoning Province, PR China.

**Keywords:** Epithelial ovarian cancer, laparotomy, peritoneal cancer index, predictive index value, long-term outcome.

## Abstract

We evaluated the ability of our two laparotomy-based models to predict optimal primary debulking surgery (PDS) and long-term outcomes of stage IIIC epithelial ovarian cancer (EOC). Data of 400 IIIC EOC patients who underwent laparotomy were retrospectively analyzed. Sensitivity, specificity, positive predictive value (PPV), negative predictive value (NPV), and overall accuracy were calculated for 10 parameters. The parameters with a specificity ≥75%, PPV ≥50%, and NPV ≥50% were included in the final predictive index value (PIV) model. Peritoneal cancer index (PCI) was calculated summarizing lesion size scores (LSSs) of 13 regions. Receiver operating characteristic (ROC) curve was used to assessed the predictive value of PIV and PCI for optimal PDS. Univariate and multivariate analyses were performed to assess the prognostic value of PIV and PCI. After PDS, 223 (55.8%) patients with RD ≤1 cm had longer progression-free survival (PFS) and overall survival (OS) than patients with RD >1 cm (PFS: 22.4 vs. 15.4 months, respectively; *P* < 0.001 and OS: 48.6 vs. 35.6 months; *P* < 0.001). PCI better predicted optimal PDS than PIV (The area under the curve of ROC: PCI 0.79 vs. PIV 0.75). The predictive value of PIV and PCI models was verified using another cohort of 77 patients. And PIV and PCI models were demonstrated to be more powerful than the published laparoscopy-based predictive index (LPS-PI) model. Patients with a PIV ≥14 were more likely to undergo suboptimal PDS with a specificity of 100%. The median PFS and OS of patients with PIV < 3 were significantly longer than patients with PIV > 3 (PFS: 19.5 vs. 16.3 months, *P* = 0.007; OS: 46.1 vs. 37.0 months, *P* = 0.009). The median PFS and OS of patients with the PCI < 17.5 were significantly longer than patients with the PCI > 17.5 (PFS: 22.9 vs. 14.5 months, *P* < 0.001; OS: 54.3 vs. 31.5 months, *P* < 0.001). PCI could better predict optimal PDS compared with PIV. PCI was an independent prognostic factor for long-term outcome of IIIC EOC patients.

## Introduction

Advanced epithelial ovarian cancer (EOC) has a poor prognosis. It is acknowledged worldwide that optimal primary debulking surgery (PDS) is a cornerstone of the treatment of advanced EOC^1^. Achievement of residual disease (RD) of <1 cm could significantly improve the outcome of patients with advanced EOC^2^, ^3^. Identification of preoperative parameters with which to predict the outcome of PDS is urgently needed.

Several studies have been performed to investigate the accuracy of preoperative radiological features and parameters of exploratory laparoscopy 4-9. As a result, models based on preoperative radiological assessment or exploratory laparoscopies were applied to filter patients with advanced EOC but the satisfactory PDS would be unavailable. However, these models were not accurate enough to predict the outcome of PDS, and they had some limitations. Although the accuracy of radiological assessment is lower than that of exploratory laparoscopy, it is difficult to estimate the lymph node status and the extent of vessel infiltration during exploratory laparoscopy. Moreover, the sample sizes in these published studies were small.

We retrospectively analyzed 400 patients with International Federation of Gynecologists and Obstetricians (FIGO) stage IIIC EOC who achieved optimal and suboptimal PDS through laparotomy. Two models based on exploratory laparotomy and patients' long-term outcomes were developed to identify the most useful parameters with which to predict optimal PDS and long-term outcomes.

## Materials and Methods

This study included patients who were treated at the Department of Gynecologic Oncology of Liaoning Cancer Hospital & Institute (Cancer Hospital of China Medical University) from January 2003 to August 2016. All enrolled patients met the following criteria: (1) diagnosis of FIGO stage IIIC EOC; (2) performance of PDS without neoadjuvant chemotherapy; (3) suspicion of intra-abdominal diffuse disease based on preoperative radiological assessments (computed tomography/ultrasonography); (4) use of platinum-based chemotherapy as postoperative adjuvant chemotherapy; and (5) availability of other data including age, preoperative serum CA125 concentration, volume of ascites, histology and grade of tumor, and Gynecologic Oncology Group performance status. The 2014 FIGO guidelines removed patients with only positive retroperitoneal lymph nodes from the definition of stage IIIC EOC 10. Therefore, we reassessed all patients' cancer stages before 2014 according to their pathological reports. All included patients provided written informed consent.

The standard surgical procedure included total abdominal hysterectomy with bilateral salpingo-oophorectomy, total omentectomy, and appendectomy. Combined multiple-organ resection, when necessary, included peritonectomy, pelvic and para-aortic lymph node dissection, resection of infiltrated bowels, resection of the infiltrated diaphragm, and partial hepatectomy or splenectomy. Optimal PDS was defined as RD of ≤1 cm, and suboptimal PDS was defined as RD of >1 cm. Moreover, the patients who were sensitive to platinum were defined as disease relapse 6 months or more after the initial platinum-based chemotherapy 11, 12. The lymph node rate (LNR) was defined as the number of positive lymph nodes divided by the total number of removed lymph nodes. Positive lymph nodes were identified by pathological examination.

### Selection of predictive parameters

Ten parameters were investigated as potential predictors of optimal PDS: infiltration of the bowel, peritoneum, diaphragm, hepatic surface, spleen, and stomach; omental caking; mesenteric retraction; and suspicion of metastasis of the pelvic and para-aortic lymph nodes. Only lesions of >2 cm were considered. We evaluated the foreshortened mesentery of the jejunum and ileum according to whether it was possible to fold back the various intestinal segments 4. The status of the pelvic and para-aortic lymph nodes was evaluated according to their size, hardness, activity, and relationship with surrounding vessels.

### Data analysis

The sensitivity, specificity, positive predictive value (PPV), negative predictive value (NPV), and overall accuracy were calculated for each parameter. Sensitivity was defined as the number of patients who were correctly identified to have RD of >1 cm (true positives) divided by the total number of patients with RD of >1 cm (true positives + false negatives). Specificity was defined as the number of patients who were correctly identified to have RD of ≤1 cm (true negatives) divided by the total number of patients with RD of ≤1 cm (true negatives + false positives). We calculated the PPV by dividing the number of true positives by the total number of positive results (true positives + false positives). We calculated the NPV by dividing the number of true negatives by the total number of negative results (true negatives + false negatives). The total accuracy was calculated as the number of true negatives plus true positives (total number of correct) divided by the total number of patients in the study.

The predictive parameters included in the model were required to meet the following criteria: specificity of ≥75%, PPV of ≥50%, and NPV of ≥50%^6^. A specificity cutoff value of 75% was chosen to minimize the number of false-positive cases (i.e., patients who in fact could achieve RD of ≤1 cm but were predicted to have RD of >1 cm). Parameters that met the above criteria were assigned a score of 1. The parameters with an overall accuracy of >60% in predicting optimal PDS were assigned an additional score of 1 to increase the number of patients whose PDS outcome was correctly identified by our model. Finally, excluding metastasis of the pelvic lymph nodes, the following parameters were assigned a score of 2: infiltration of the stomach (>2 cm), bowel (>2 cm), peritoneum (>2 cm), diaphragm (>2 cm), hepatic surface (>2 cm), and spleen (>2 cm); omental caking; mesenteric retraction; and metastasis of the para-aortic lymph nodes (suspected infiltration of vessels)** (Table 1)**.

**Figure 1:**
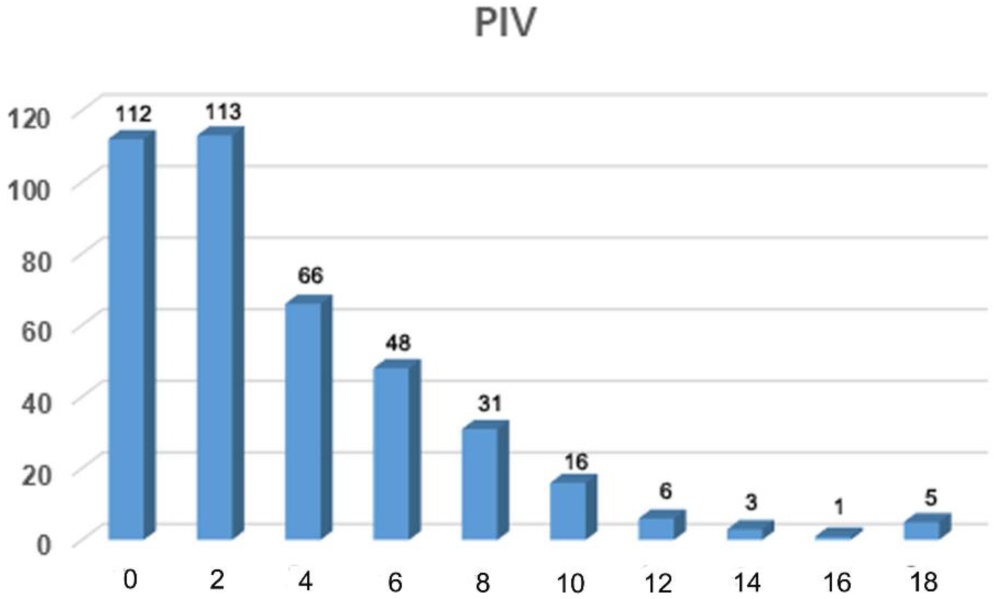
Frequency distribution of predictive index value for 400 patients with FIGO stage IIIC epithelial ovarian cancer.

A total predictive index values (PIVs) of each patient was calculated using the above-described scoring system **(Figure 1)**. The sensitivity, specificity, PPV, NPV, and accuracy were tabulated for each PIV of 0 through 18** (Table 2)**. Receiver operating characteristic (ROC) curve analysis was used to assess the capacity of the PIV model to predict the patients who could achieve optimal PDS 13.

Peritoneal Cancer Index (PCI) model was used to assess the abdominal and pelvic dissemination of gastrointestinal cancer 14. The pelvis and abdomen were divided into 13 regions. The Lesion Size Scores (LSSs) of each region was assessed respectively. Each LSS ranged from 0 to 3: LSS 0 referred to no tumor; LSS 1 referred to the size of tumor less than 0.5cm; LSS 2 referred to the size of tumor less than 5cm; and LSS 3 referred to the size of tumor more than 5cm. In this retrospective study, we constructed a laparotomy-based PCI model through summarizing all the LSSs from 13 regions.

Progression-free survival (PFS) and overall survival (OS) were analyzed using Kaplan-Meier method. Univariate analyses were performed using log-rank tests. And multivariate analyses were performed using Cox regression analysis to assess the effects of the prognostic factors, which were expressed as hazard ratios (HRs). *P* < 0.05 was considered statistically significant, and all *P* values were two-sided. All data analyses were performed by SPSS 16.0 (SPSS Inc., Chicago, IL).

## Results

In total, 400 patients with FIGO stage IIIC EOC and median follow-up period of 85.4 months were included in this analysis as development cohort. Another 77 patients with the same baseline were analyzed as validation cohort. The patients' characteristics are listed in **Table S1**.

### Long-term effect of Residual Disease

In total, 223 (55.8%) of 400 patients achieved optimal PDS with RD of ≤1 cm, in which 121(30.3%) patients without RD. The median PFS and OS of the patients who achieved optimal PDS were significantly longer than patients with RD of >1 cm (PFS: 22.4 vs. 15.4 months, respectively; *P* < 0.001 and OS: 48.6 vs. 35.6 months, respectively; *P* < 0.001) **(Figure S1A and S1B)**.

### Construction of PIV model

The status of the pelvic lymph nodes was not included as a predictive parameter because of its low NPV (49%), and no predictive index score was assigned. However, infiltration of the bowel (>2 cm), peritoneum (>2 cm), diaphragm (>2 cm), hepatic surface (>2 cm), spleen (>2 cm), and stomach (>2 cm); omental caking; mesenteric retraction; and metastasis of the para-aortic lymph nodes (suspected infiltration of vessels) satisfied the inclusion criteria **(see Methods)**. A predictive index score of 1 was subjectively assigned to each parameter. The parameters with an overall accuracy of ≥60% were assigned an additional score of 1 and a final predictive index score of 2 **(Table 1)**.

The PIVs for each of the 400 patients were tabulated using this predictive scoring system according to the status of the inclusive parameters and ranged from 0 to 18 points **(Figure 1)**. The frequency distribution of individual PIVs showed the largest concentration of patients in the group with a PIV of 2 (n = 113). The sensitivity, specificity, PPV, NPV and accuracy of each PIV of 0 through 18 are shown in **Table 2**. Both the specificity and PPV of patients with a PIV of ≥18 were 100 **(Table 2)**, suggesting that these patients had a very low possibility of achieving optimal PDS.

**Figure 2:**
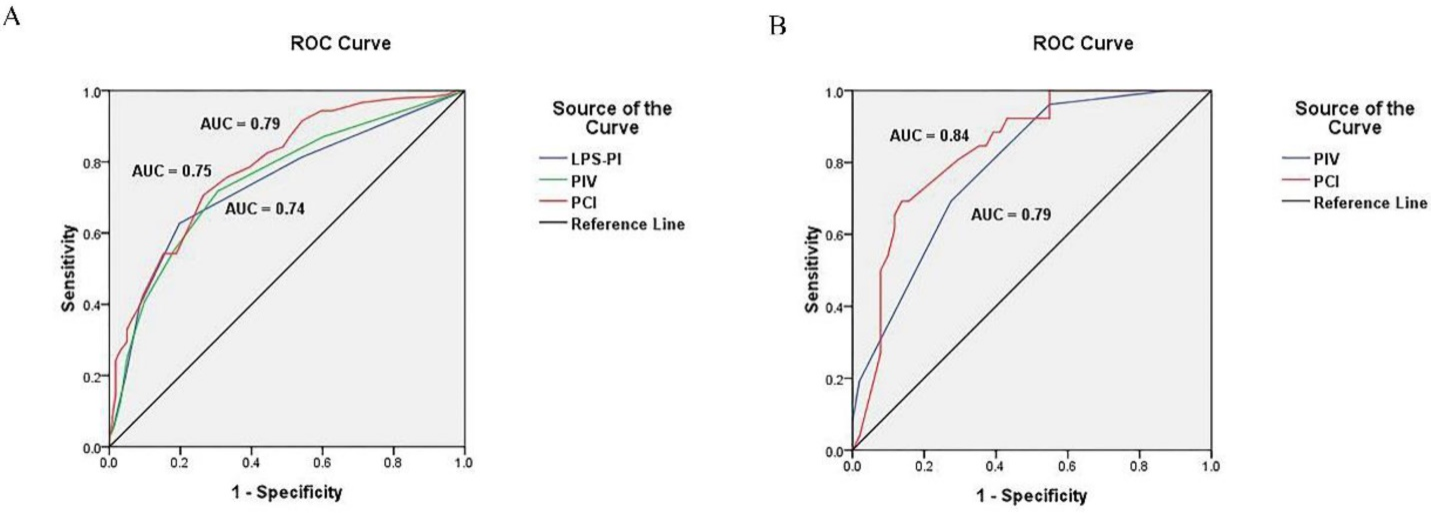
**Receiver operating characteristic curve of models. (A)** ROC curve of PIV, PCI and LPS-PI model in development cohort; **(B)** ROC curve of PIV and PCI model in validation cohort.

**Figure 3:**
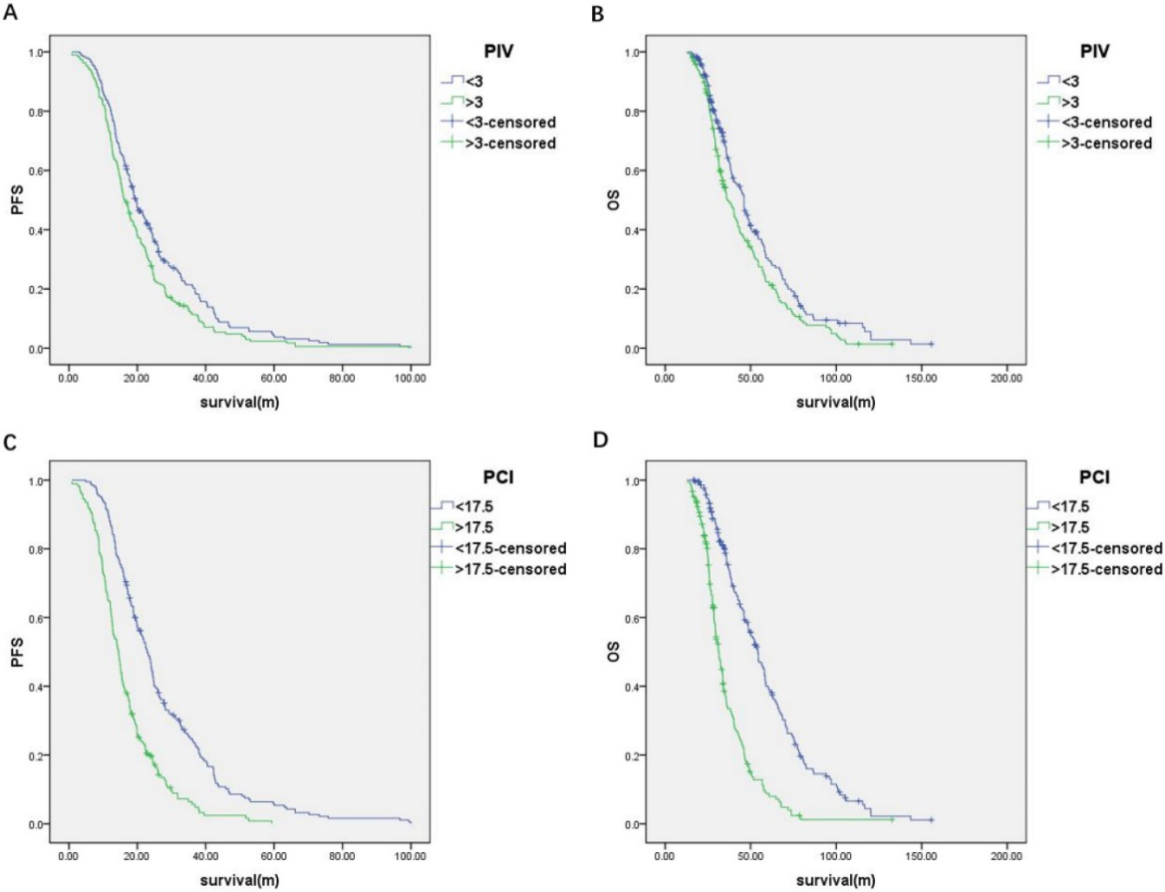
**(A)** Progression-free survival and **(B)** overall survival of the patients classified by PIV; **(C)** Progression-free survival and **(D)** overall survival of the patients classified by PCI.

The predictive value of the PIV model to ideal PDS was assessed by ROC curve analysis. The area under the curve (AUC) was 0.75 [95% confidence interval (95% CI), 0.70-0.80; *P* < 0.001] (**Figure 2**). The cut-off value of PIV was 3. The median PFS and OS of the patients with the PIV < 3 were significantly longer than patients with the PIV > 3 (PFS: 19.5 vs. 16.3 months, *P* = 0.007; OS: 46.1 vs. 37.0 months, *P* = 0.009) **(Figure 3A and 3B)**. Furthermore, the predictive value of the PIV model to ideal PDS was verified in another cohort of 77 patients enrolling from September 2016 to December 2017 in our hospital. The AUC was 0.79 (95%CI, 0.69 to 0.89) (**Figure 2**).

### Construction of PCI model

The PCI for each of the 400 patients were calculated through summarizing all the LSSs from 13 regions **(see Methods and Table S2)**. The predictive value of the PCI model to optimal PDS was assessed by ROC curve analysis. The AUC was 0.79 (95% CI, 0.74-0.83; *P* < 0.001) (**Figure 2**). The cut-off value of PCI was 17.5. The median PFS and OS of the patients with the PCI < 17.5 were significantly longer than patients with the PCI > 17.5 (PFS: 22.9 vs. 14.5 months, respectively; *P* < 0.001 and OS: 54.3 vs. 31.5 months, respectively; *P* < 0.001) **(Figure 3C and 3D)**. Furthermore, the predictive value of the PCI model to ideal PDS was verified in another cohort of 77 patients enrolling from September 2016 to December 2017 in our hospital. The AUC was 0.84 (95%CI, 0.75 to 0.93) (**Figure 2**).

We also calculated the score for each patient according to the published laparoscopy-based predictive index (LPS-PI) model 5. The author assigned a score of 2 to all six parameters: omental cake, peritoneal carcinomatosis, diaphragmatic carcinomatosis, bowel infiltration, stomach infiltration and liver metastases. And the predictive value of the LPS-PI model to ideal PDS was valued, which was not better than PIV or PCI model. The AUC of LPS-PI was 0.74 (95%CI, 0.69 to 0.79) (**Figure 2**).

### PCI was an independent prognostic factor

Regarding PFS, from univariate analysis the variables associated with a shorter PFS included volume of preoperative ascites, preoperative CA125 value, histology of tumor, RD, PIV and PCI. Furthermore, from multivariate analysis by Cox regression the independent variables associated with a shorter PFS were volume of preoperative ascites, histology of tumor, RD and PCI **(Table 3)**.

Regarding OS, from univariate analysis the variables associated with a shorter OS included volume of preoperative ascites, preoperative CA125 value, histology of tumor, RD, blood loss during PDS, blood transfusion, PIV and PCI. Multivariate analysis by Cox regression showed that the independent variables associated with a shorter OS were volume of preoperative ascites, histology of tumor and PCI **(Table 3)**.

## Discussion

This retrospective study included 400 patients from a Chinese cancer hospital and involved strong and active cooperation among multiple departments. We generated two models in which PCI model could better predict the optimal PDS of IIIC EOC patients than PIV model. These two models were based on laparotomy rather than laparoscopic or radiological features. The results indicated that IIIC EOC patients with a PIV >3 or a PCI >17.5 had a very low possibility to achieve optimal PDS. This suggested that such patients should not be selected for PDS.

Advanced EOC is characterized by intra-abdominal diffusion of disease. Therefore, the predictive evaluation of a model generated by multiple parameters could be stronger than the use of a single parameter and might be able to reflect the biological aggressiveness and burden of the tumor. By calculating the sensitivity, specificity, PPV, and NPV, we identified the following predictive parameters of optimal PDS for the first time: infiltration of the bowel, peritoneum, diaphragm, hepatic surface, spleen, and stomach; omental caking; mesenteric retraction; and metastasis of the para-aortic lymph nodes (suspected infiltration of vessels). We then tabulated the PIV of each patient and generated a PIV model according to all parameters. The specificity and PPV of this multiple-parameter model were better than those of each single parameter.

Whether PDS for treatment of advanced EOC could be optimal depends on the total capacity of the oncologic group. The rate of optimal PDS reached 55.8% in the present study, which is higher than in most other Chinese institutions. Moreover, this percentage is similar to that in reports from the United States and Europe 1-3.

This study has several strengths. A total of 400 patients with FIGO stage IIIC EOC were evaluated. All of the patients achieved laparotomy. The use of laparotomy overcame the limitations of inaccessible lymph nodes and the lack of tactile sensation associated with laparoscopy. Additionally, this approach is more accurate than radiological prediction. Our retrospective analysis allowed us to identify comprehensive parameters and generate a PIV model. In the future, we plan to revise our laparoscopic predictive system based on our comprehensive retrospective analysis. This study also had some limitations. Firstly, the capacity of upper abdominal surgery in our situation has developed since 2013. So bias existed between the patients treated before and after 2013. Secondly, the status of BRCA 1/2 mutation was not available for the patients in this study. Thirdly, the low rate of lymph nodes dissection declined the validity of PIV model. Lastly, the main one being its retrospective nature. A prospective study in our institution is ongoing.

In conclusion, optimal PDS significantly prolonged the PFS and OS of patients with FIGO stage IIIC EOC. Infiltration of the bowel, peritoneum, diaphragm, hepatic surface, spleen, and stomach; omental caking; mesenteric retraction; and metastasis of the para-aortic lymph nodes (suspected infiltration of vessels) were predictive parameters for optimal PDS. Our PIV based on these multiple parameters and PCI model could preferably predict RD. The opportunity of the patients with a PCI >17.5 or a PIV > 3 to achieve optimal PDS was very small. Considering the prognostic effect of the biological behavior of a tumor, investigation of accurate indicators and predictors of the malignancy of EOC should continue.

## Supplementary Material

Supplementary figures and tables.

## Figures and Tables

**Table 1:** PIV model.

parameter	Sensitivity (%)	Specificity (%)	PPV (%)	NPV (%)	Accuracy (%)	score
bowel (>2cm)	58	75	65	69	68	2
peritoneum (>2cm)	51	78	65	67	66	2
diaphragm (>2cm)	36	87	70	63	65	2
Hepatic surface (>2cm)	17	96	75	59	61	2
spleen (>2cm)	12	97	76	58	60	2
gastric (>2cm)	8	98	75	57	60	2
Omental cake	41	77	59	62	61	2
mesenteric retraction	16	92	62	58	60	2
Para-aortic lymph nodes	17	92	63	58	60	2
Pelvic lymph nodes	8	93	57	49	49	0

Abbreviations: PPV, positive predictive value; NPV, negative predictive value.

**Table 2:** Predictive Index Value Model.

PIV	Sensitivity (%)	Specificity (%)	PPV (%)	NPV (%)	Accuracy (%)	Inappropriate lack of exploration (%)	Unnecessary exploration (%)
0	87	40	53	79	61	47	21
≥2	68	76	69	75	73	31	25
≥4	47	88	75	68	70	25	32
≥6	27	94	77	62	64	23	38
≥8	13	97	77	58	60	23	42
≥10	6	99	79	57	58	21	43
≥12	4	100	88	57	57	12	43
≥14	3	100	100	56	57	0	44
≥16	2	100	100	56	57	0	44

Abbreviations: PPV, positive predictive value; NPV, negative predictive value.

**Table 3:** Univariate and multivariate analyses.

	No. of patients	PFS			OS		
		Univariate analysis, *P*	Multivariate analysis, *P*	HR(95%CI)	Univariate analysis, *P*	Multivariate analysis, *P*	HR(95%CI)
Age (y)							
<65	339	Referent			Referent		
≥65	61	0.995			0.552		
Pre-operative ascites volume (ml)							
<1000	169	Referent	Referent		Referent	Referent	
≥1000	231	0.001	0.003	1.40(1.12-1.74)	0.006	0.022	1.32(1.04-1.68)
Pre-operative CA125 value (U/ml)							
<1000	290	Referent	Referent		Referent		
≥1000	110	0.090	0.899		0.225		
Histology							
HGSOC	31	Referent	Referent		Referent	Referent	
Non-HGSOC	369	0.061	0.038	0.67(0.46-0.98)	0.088	0.034	1.55(1.04-2.31)
Stay in hospital (d)							
≤30	330	Referent			Referent		
>30	70	0.113			0.267		
RD (cm)							
≤1	223	Referent	Referent		Referent	Referent	
>1	177	<0.001	<0.001	1.59(1.23-2.06)	<0.001	0.417	
Surgical procedure (min)							
≤240	331	Referent			Referent		
>240	69	0.408			0.436		
Blood loss during PDS (ml)							
<400	155	Referent			Referent	Referent	
≥400	245	0.656			0.035	0.807	
Blood transfusion							
No	140	Referent			Referent	Referent	
Yes	260	0.193			0.023	0.572	
PIV							
<3	205	Referent	Referent		Referent	Referent	
>3	195	0.007	0.992		0.009	0.706	
PCI							
<17.5	216	Referent	Referent		Referent	Referent	
>17.5	184	<0.001	<0.001	1.82(1.43-2.30)	<0.001	<0.001	2.61(2.00-3.39)

Abbreviations: HGSOC, high grade serous ovarian cancer; PIV, predictive index value; PCI, peritoneal cancer index; PFS, progression free survival; OS, overall survival; HR, hazard ratio; RD, residual disease; PDS, primary debulking surgery.
