# Supplementary material for: Role of Laparotomy-based Parameters in Assessment of Optimal Primary Debulking Surgery and Long-term Outcomes in Patients with Stage IIIC Epithelial Ovarian Cancer
Source: J Cancer. 2020 Jan 1;11(4):983–9. doi: 10.7150/jca.32317 (PMC6959015; doi:10.7150/jca.32317)
Supplement: Supplementary file 1 — Supplementary figures and tables. [file jcav11p0983s1.pdf]

Table S1. Patients' characteristics

Variable	Development cohort	Validation cohort
Number of patients	400	77
Size of RD		
RD=0cm	121 (30.3%)	20 (26.0%)
RD≤1cm	102 (25.5%)	31 (40.3%)
RD>1cm	177 (44.2%)	26 (33.7%)
Age	54.4±8.9	53.8±7.8
GOG performance status		
0-1	323(80.7%)	55 (71.4%)
2	66(16.5%)	18 (23.4%)
3	11(2.8%)	4 (5.2%)
Preoperative ascites volume (ml)	2600±1680.6	2400±1540.9
Preoperative CA125 level (U/ml)	3300±689.5	3100±567.9
Grade of tumor		
1	27(6.8%)	3 (3.9%)
2	144(36.0%)	25 (32.5%)
3	229(57.2%)	49 (63.6%)
Histotype of tumor		
Serous	259(64.8%)	59 (76.6%)
Endometriod	47(11.7%)	8 (10.4%)
Muscinous	4(1.0%)	1 (1.3%)
Clear cell	5(1.3%)	2 (2.6%)
Adenocarcinoma, not otherwise specified	85(21.2%)	7 (9.1%)
Status of lymph nodes		
Negative	21(5.3%)	12 (15.6%)
Positive	45(11.2%)	20 (26.0%)
Unknown	334(83.5%)	45 (58.4%)
Median PFS (months)	17.8 (12.3-26.0)	-
Median OS (months)	34.2 (26.0-52.5)	-

Abbreviations: RD, residual disease; GOG, Gynecologic Oncology Group; PFS, progression-free survival; OS, overall survival

Table S2. PCI model

region	LSS 0	LSS 1	LSS2	LSS 3
Central	0	1	2	3
Right Upper	0	1	2	3
Epigastrium	0	1	2	3
Left Upper	0	1	2	3
Left Flank	0	1	2	3
Left Lower	0	1	2	3
Pelvis	0	1	2	3
Right Lower	0	1	2	3
Right Flank	0	1	2	3
Upper Jejunum	0	1	2	3
Lower Jejunum	0	1	2	3
Upper Ileum	0	1	2	3
Lower Ileum	0	1	2	3

Notes: LSS 0 referred to no tumor; LSS 1 referred to the size of tumor less than 0.5cm; LSS 2 referred to the size of tumor less than 5cm; and LSS 3 referred to the size of tumor more than 5cm.

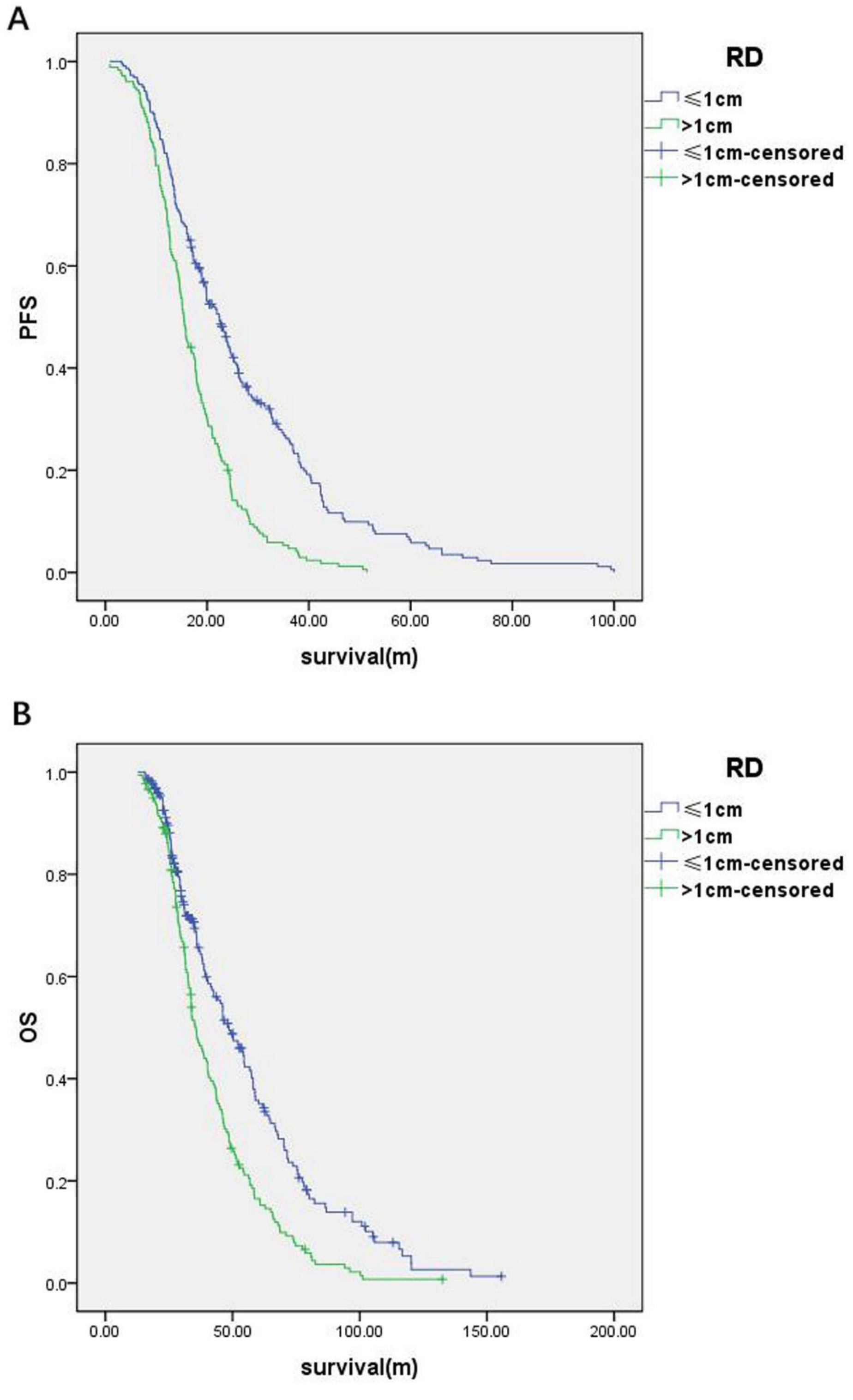

Figure S1. Progression-free survival and overall survival of patients with residual disease of ≤1 cm and >1 cm.
